# The Effects of Sudan's Armed Conflict on Economy and Health: A Perspective

**DOI:** 10.1002/hsr2.70424

**Published:** 2025-02-06

**Authors:** Esraa Mahadi Ali Mohamed, Don‐Eliseo Lucero‐Prisno

**Affiliations:** ^1^ University of Medical Sciences and Technology Khartoum Sudan; ^2^ Department of Non‐Communicable Disease Epidemiology London School of Hygiene and Tropical Medicine London UK

## Abstract

Sudan's economy has been greatly affected by the armed conflict through 15 different channels at both micro and macro levels. The requested fund to save live of sudanese population is 2.6 billion US$ from which only 33% was allocated. The humanitarian crisis in Sudan has lead to contraction in economy and thus agriculture, health, water supply, education, and bankig which all resulted in increased mortality and morbidity rates, food insecurity, violation in human rights and inflation. There is a rising humanitarian need for assistance, in addition to urgent interventions to stop war and cease fire. International law must be enforced through international organizations, the voice of Sudanese civil society must be coordinated if not united, and several measures must be taken after the conflict resolved to address the impacts of conflict.

## 1 Background

Armed conflict is affecting Sudan's economy at both macro and micro levels through 15 different channels including: and imports and exports (agriculture), production, industries (mining and construction), services (public and private), and also the foreign financial transactions for households [1]. Sudan's economy has been declined as a result of armed conflict as the growth forecast went downward by 12.5% which when compared to Syria and Yemen (5% per year); this percent seemed to be very alarming [2].

The 2023 Sudan Humanitarian Response Plan (HRP) illustrated that the required fund to cover the lifesaving needs for 18.1 million population is US$2.6 billion, from which only 33.4% (US$ 856.2 million) has been allocated by November 15th [3]. The Sudan's Gross Domestic Product (GDP) is estimated to lower by 48% by the end of this year, from which the economical losses are estimated to be US$ 15 billion. The impact of war on national economy and individual sectors will result in increase of poverty by around 1.8 million people to reach 39.3 million in total. The total loss of jobs was 5.2 million from which 2.7 million were in services sector. The economic activity has been disrupted in Khartoum and other states of Sudan due to the violent conflict [4].

The humanitarian crisis and economic contraction resulted from the armed conflict have greatly affected populations' lives. Millions of people have been forced to flee their homes and escape from the risks, threats, and violence. There is an increased prevalence of communicable diseases and lack of access to clean water and health services, malnutrition as a result from the disrupted food chains and food insecurity, human rights violations and sexual violence, inflation and reduced income, destruction of banking systems, and disruption of the education systems since the schools have been destroyed and teachers have been enforced to displace. The number of people killed in Sudan's armed conflict is at least 15,500, and some estimates stated that not less than 150,000. Measles outbreaks killed over 1000 child, and cholera outbreaks threaten lives of 11,000 as a result of low immunization coverage [5]. There were also confirmed cases of dengue fever [6].

## 2 Impacts on Economy and Health

### 2.1 Agriculture

The households' income from agriculture has been declined and disrupted due to the poor preparation of the agriculture season. Agriculture constitutes a major source of income at the rural areas and represents more than 39% of the total income. Food insecurity will be expected to affect more than 20.3 million population and the mortality rates will be more than 700,000 child as a result of malnutrition that will occur; as a result of shutdown of more than 400 establishments in Khartoum due to vandalism, also, the reduction of yields and production will happen due to the shortage of inputs like fertilizers, fuel, and seeds. Essential food commodities became unaffordable for an increasing number of households due to the hike in inflation and increase in prices [6].

### 2.2 Health

Sudan's already fragile health system is about to undergo a complete collapse after the war started. In Khartoum, those who were suspected to violence, injured, or chronically ill patients couldn't have access for treatment because the emergency rooms are either out of service, or congested, or the medications are unavailable; given the fact that most of Khartoum pharmacies are closed. The impact went further indirectly for those who are unaffected by the violence directly; they are suffering from chronic shortages of pharmaceuticals because the pharmacies are either run out of supplies and this is due to the unfunctioning medical companies at Khartoum state which was considered as the main supply for the other states', the very high prices of medicines due to the increased demand and limited supply and also the very high inflation rate of the hard currency making medications unaffordable. Even for those who have easier access, they are displaced at schools, and camps which are overwhelmed and they suffer from preventable diseases, like cholera and malaria and die; as a result of absence of immunization and catch up campaigns [7].

Rapid Support Forces (RSF) have seized many public health assets that are critical for health services delivery and National Medical Supplies Fund (NMSF) was one of them which resulted in limited access to medical supplies. The actual cost of war is not about the current losses for civilians but the long‐term impact it will leave on the health system [8]. More than 70% of hospitals ran out of service due to the power outages and shortages of fuel, lack of medical supplies, and critical lack of health workforce. The impact of medications delivery couldn't be accurately estimated; and this is due to the country's weak health information, monitoring and evaluation systems. However, life‐saving drugs for dialysis, cancer, trauma, obstetric and child care, and emergency are severely disrupted. About 1 million Sudanese refugees and 3.2 million internally displaced are suffering from access to health services and medical supplies. The public health sector is chronically underfunded and its financial losses are more than US$ 700 million, where its GDP went down by 1.4% in which the funds have been mobilized for the military and defence functions. All of these factors make it very difficult to achieve Universal Health Coverage (UHC) [8]. Out of total government budget, 11% was lost in health sector and 1.7 million children under‐one loss lifesaving vaccines [6].

In Khartoum state, around 19% of Sudan's population are living there where‐also more than one‐third of the health services are provided; and this is unfortunately due to the centralized system. A severe economic standstill has witnessed Khartoum and other states of Sudan due to the blocking of internal trade routes which resulted in unavailability of goods and services, food and safe water, and pharmaceuticals. The power outages, and the inability to refuel generators led to loss of 40 million vaccines and insulin vials [9]. At the other states of Sudan, such as Elgenina, all of the medical centers are inoperable due to the looting, attacks, and dwelling of them and also the drugs outlets and doctors dwellings. The only renal center at Elgenina is currently out of service. MSF declared that their offices in Nyala and Darfur states have been broken where they store their medical supplies and equipments where also their cars and other supplies were taken [9].

### 2.3 Education

Regarding the education sector, the estimated cost of education loss is equivalent to US$ 26 billion, as the schools were closed and teachers were not receiving their salaries, and the school buildings were become emergency shelters due to the lack of funds to host the Internally Displaced Population (IDP) [6].

### 2.4 Water Supply

Destruction of water supply facilities has resulted in acute water shortages. Water development financing through the federal government, which supposed to be 14% from the total capital transfers has stopped for all areas [6].

## 3 Conclusion, Recommendations, and Policy Implications

The rising humanitarian needs must be addressed immediately through provision of economic services and cash assistance, secure shelters, health and nutrition services, water, sanitation, and hygiene programs, and women protection and empowerment services [5].

This perspective has been written to shed lights about Sudan's catastrophe to point the levels of how political crisis deteriorates economy and how they impact the health system function, so it can help bringing awareness of decision‐makers and global leaders for more actions and aids and prevent this from evolving. We and all healthcare professionals in Sudan ‐those who are directly affected by the war and those who are responsible of all Sudanese health systems‐ are strongly disagree with what is currently happening between Sudanese Armed Forces (SAF) and RSF since it is an internal affair and it could have been solved in a better way other than displacing civilians, letting them leaving their homes, losing their assets and savings that they have been working for hardly during their whole lives, losing their jobs, having difficulties of getting access to live necessities including food, water, electricity, shelter, and treatment. We implore if they don't want to stop the war until one part took the full control, they must agree for long‐term cease fire to open routes for access for life‐saving commodities and allow humanitarian aid delivery because now the delivered amount is only 53 trucks which represent only one‐third of the planned amount [10].

Also, global community leaders and neighboring countries must not forget about Sudan as they must advocate for further negotiations to stop war and cease fire and holding both sides of war accountable for the happening human rights violations. We loudly calling WHO, UN agencies, World Bank, and all international organizations, donors, and entities to report regularly and provide solid health information system, provide more lifesaving support, including oxygen, blood, and medications to improve the accessibility to public health services and fasten rehabilitation. Furthermore, provision of training for citizens about infectious diseases, community mobilization and access to shelters, and essential life support are all urgently required. More collaboration, cooperation, and partnerships are needed with academic institutions and researchers of healthcare workers to generate evidence‐based data and research works on the impact of war on vulnerable groups including: women and children, elderly, refugees, and Internally Displaced Population (IDP) [10].

International law must be enforced through international organizations, which play a crucial role and have a significant impact in conflict resolution, filing lawsuits against perpetrators of crime, and protecting civilians. Several measures can be taken through the United Nations including: sending mediation or negotiation missions, resolutions represented by violence cessation and sanctions imposition, which will exert economic and political pressure on the parties of the war [11].

Furthermore, the voice of Sudanese civil society must be coordinated if not united, before the initiation of the political process that is facilitated by external mediators, and must also be an engagement of youth in decision making process. The role of the military must be clear at this period so as to bridge the gaps. Political actors should discuss their points of convergence and divergence in meetings, or workshops to address the issues of misunderstanding and mistrust and facilitate the process of consensus building [12].

When conflict resolved, substantial resources will be required to address the impacts of conflict. This including fiscal support for households, financing for enterprises, increased public investment for infrastructure repair, and provision of incentives for businesses. However, many interventions should be considered for resources mobilization and spend on education, health, water, creating public financing schemes for the poor, strengthen social protection through banking and payment system reform, which are all will directly concern the well‐being of population and children [6].

Those measurements are including: fiscal federalism system strengthening, avoid the inflation that could happen if the government conduct monetization of the inevitable fiscal deficit in 2023, Bank of Sudan and all of the commercial banks should develop a plan for business continuity and disaster recovery, improve the focus on digitization and financial inclusion, reform and make water corporations sustainable, community inclusion in designation of social protection and resilience programs, and regularize the electrical supply through substantial investments in solar energy [6].

At the end, we call all the global health communities to make the voices of Sudanese heard by all of the world, and maintaining focus on Sudan's conflict as the Sudanese citizens have the right of health for all despite the ongoing conflict [10].

## Author Contributions

**Esraa Mahadi Ali Mohamed:** writing–original draft. **Don‐Eliseo Lucero‐Prisno:** supervision.

## Conflicts of Interest

The authors declare no conflicts of interest.

## Transparency Statement

The lead author Esraa Mahadi Ali Mohamed affirms that this manuscript is an honest, accurate, and transparent account of the study being reported; that no important aspects of the study have been omitted; and that any discrepancies from the study as planned (and, if relevant, registered) have been explained.

## Data Availability

The authors have nothing to report.
